# Kernel size‐related genes revealed by an integrated eQTL analysis during early maize kernel development

**DOI:** 10.1111/tpj.14193

**Published:** 2019-01-25

**Authors:** Junling Pang, Junjie Fu, Na Zong, Jing Wang, Dandan Song, Xia Zhang, Cheng He, Ting Fang, Hongwei Zhang, Yunliu Fan, Guoying Wang, Jun Zhao

**Affiliations:** ^1^ Biotechnology Research Institute Chinese Academy of Agricultural Sciences Beijing 100081 China; ^2^ Institute of Crop Sciences Chinese Academy of Agricultural Sciences Beijing 100081 China

**Keywords:** expression quantitative trait loci, complex trait, m^6^A RNA modification, kernel development, *Zea mays* L

## Abstract

In maize, kernel traits strongly impact overall grain yields, and it is known that sophisticated spatiotemporal programs of gene expression coordinate kernel development, so advancing our knowledge of kernel development can help efforts to improve grain yields. Here, using phenotype, genotype and transcriptomics data of maize kernels at 5 and 15 days after pollination (DAP) for a large association mapping panel, we employed multiple quantitative genetics approaches—genome‐wide association studies (GWAS) as well as expression quantitative trait loci (eQTL) and quantitative trait transcript (QTT) analyses—to gain insights about molecular genetic basis of kernel development in maize. This resulted in the identification of 137 putative kernel length‐related genes at 5 DAP, of which 43 are located in previously reported QTL regions. Strikingly, we identified an eQTL that overlaps the locus encoding a maize homolog of the recently described m^6^A methylation reader protein ECT2 from Arabidopsis; this putative ^epi^eQTL is associated with 53 genes and may represent a master epi‐transcriptomic regulator of kernel development. Notably, among the genes associated with this ^epi^eQTL, 10 are for the main storage proteins in the maize endosperm (zeins) and two are known regulators of zein expression or endosperm development (*Opaque2* and *ZmICE1*). Collectively, beyond cataloging and characterizing genomic attributes of a large number of eQTL associated with kernel development in maize, our study highlights how an eQTL approach can bolster the impact of both GWAS and QTT studies and can drive insights about the basic biology of plants.

## Significance Statement

Quantitative genetics analysis of genomic, transcriptomic and phenotypic data of a large association panel of maize inbred lines allowed us to identify and characterize trends among eQTL regions associated with kernel traits and the complex spatiotemporal programs of gene expression that impact maize kernel development. Fascinatingly, we found an eQTL region overlapping the locus encoding the maize ortholog of the very recently described m^6^A reader protein ECT2 from *Arabidopsis*.

## Introduction

Maize (*Zea mays* L.) is not only of worldwide importance as a food, feed and source of industrial products, but is also a long‐influential biological model species that exhibits extensive phenotypic and genetic diversity (Hake and Ross‐Ibarra, 2015). Its kernels, like other seeds, are the primary storage organs that provide essential components for plant growth and development (Scanlon and Takacs, 2009). The number of harvested kernels and their individual kernel size—two complex genetic traits that massively influence yield—result from the multi‐step and tightly regulated processes of kernel development (Li *et al*., 2013b; Chen *et al*., 2014; Doll *et al*., 2017). Thus, advancing our knowledge of the genetic and even molecular genetics mechanisms underlying kernel development will have large impacts for ongoing global efforts seeking to optimize maize grain yield.

Association mapping, also known as linkage disequilibrium (LD) mapping, exploits recombination events in natural populations to harness genetic diversity information to potentially resolve phenotypic variation for complex traits down to the level of single genes or even individual nucleotides. Different from genome‐wide association studies (GWAS) in humans, GWAS in crop plants are typically based on a permanent resource often known as an association panel—a population of diverse (and preferably homozygous) genetic lines that only needs to be genotyped once but can be re‐phenotyped for many traits. Maize, owing in part to its out crossing nature and attendant rapid LD decay (within ∼2 kb), as well as its great genetic diversity, is an important model for the ongoing development of powerful GWAS methods (Huang and Han, 2014). Nevertheless, researchers using GWAS must consider and manage the known massive influence of population structure in driving both increased false‐negative rates and decreased false‐positive rates. Indeed, even with careful management, it remains the case that some genuinely influential loci will not be detected (false‐negatives) in GWAS of association panels because quantitative trait loci (QTL) allele distribution is correlated with population structure (Bazakos *et al*., 2017).

One interesting conceptual approach that can help improve the scope of large quantitative genetics studies is known as expression quantitative trait loci (eQTL) analysis. This increasingly popular method is based on conducting association analysis between gene expression levels and genetic variants, and it can identify genetic polymorphisms that affect gene expression levels; it is also helpful for building links between regulators and targets when constructing gene regulatory networks (Hansen *et al*., 2008; Kliebenstein, 2009; Hammond *et al*., 2011). Furthermore, eQTL signals can also reveal the local and distant trait‐associated variants in GWAS studies, thereby linking both expression data and DNA sequence variations to phenotypes and addressing complex traits (Potokina *et al*., 2008; Li *et al*., 2013b; Albert and Kruglyak, 2015). Importantly, there have also been demonstrations that integration of eQTLs with quantitative trait transcript (QTT) analyses can facilitate the identification of causative genes for complex traits (Passador‐Gurgel *et al*., 2007; Petretto *et al*., 2008).

It is now appreciated that a sequence of key ultimately yield‐related developmental processes (e.g. syncytium formation, cellularization, cellular differentiation, etc.) occur during the early period of maize kernel development [from pollination up to about 15 days after pollination (DAP); Sabelli and Larkins, 2009]. It is also known that this phase of development is regulated by sophisticated spatiotemporal programs of gene expression (Sekhon *et al*., 2011; Li *et al*., 2014; Doll *et al*., 2017). Here, using phenotype, genotype and transcriptomics data of kernels at 5 and 15 DAP for a large association mapping panel comprised of tropical, subtropical and temperate inbred lines, we conducted eQTL, QTT and GWAS analyses, and integrated the results to gain insights about the genetic and molecular genetic basis of kernel development in maize. We noted several interesting trends relating to development‐stage‐specific eQTLs and the distances of these regulatory regions to their associated genetic loci. We also found an eQTL that overlaps the locus encoding the maize homolog of the recently described m^6^A methylation reader protein ECT2 from Arabidopsis; this putative ^epi^eQTL is associated with 53 genes and apparently regulates the metabolism of the primary protein storage proteins in maize kernels. Beyond underscoring the utility of eQTL analysis for identifying multi‐level mechanisms of gene regulation for complex traits that would be missed by genome‐focused GWAS and missed by transcriptome‐focused QTT analysis, our identification of a putative ^epi^eQTL for kernel traits overlapping the maize *ECT2* locus illustrates how quantitative genetics approaches can lead to basic biological insights.

## Results and Discussion

### **Genotyping of a maize association panel and expression quantitative trait loci analysis of genes expressed in kernels at 5 DAP**

**Figure 1:**
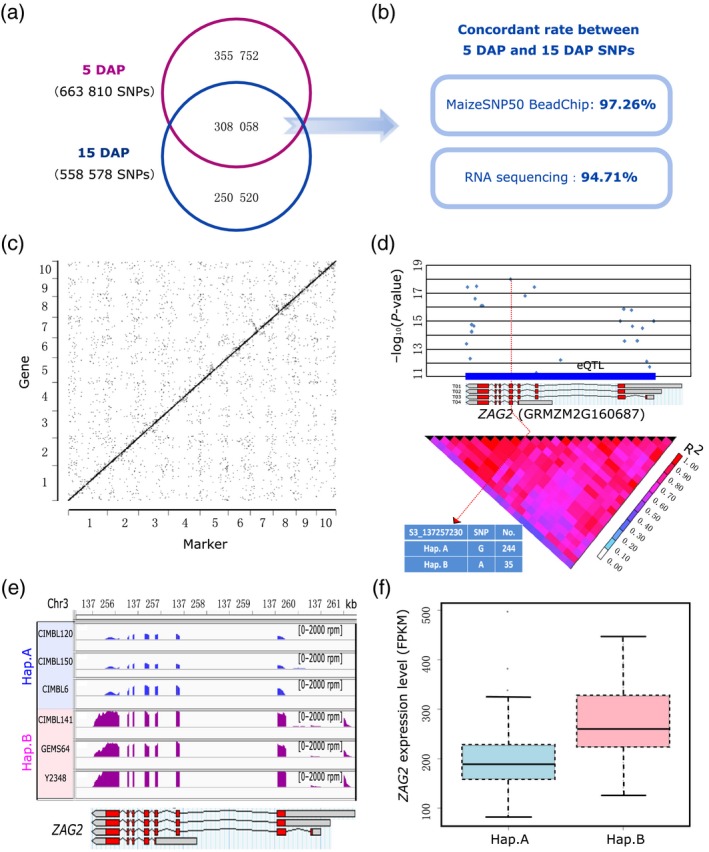
Overview of single nucleotide polymorphisms (SNPs) and expression quantitative trait loci (eQTL) results for kernels at 5 days after pollination (DAP). (a) The number of SNPs called from 5 DAP and 15 DAP (Fu *et al*., 2013), and their overlap. (b) Concordance rate of the SNPs shared by the two developmental stages. (c) The start position of the 18 377 eQTL‐associated genes (*y*‐axis) against the position of the most significant SNPs in their eQTL regions (*x*‐axis). (d) Display of the eQTL results for the *ZAG2* gene as an example. The upper panel shows the position of the eQTL and the association signals of all the SNPs located in this eQTL region, while the linkage disequilibrium (LD) plot for all the SNPs included is shown in the lower panel. Information for the most significant SNP is highlighted in the blue box. (e, f) The expression divergence for different haplotypes based on the most significant SNP in *ZAG*2. (e) Three random samples were chosen for each haplotype, and the abundance of mapped reads is shown for the same coverage range. (f) The overall expression divergence of the two haplotypes of *ZAG2* displayed as a box plot.

The uniquely mapped reads from the RNA‐seq data of 5‐DAP kernels were used for single nucleotide polymorphism (SNP) calling and genotyping of each of the 282 maize inbred lines (Table S1). After quality control and imputation, we identified a total of 663 810 high‐quality SNPs. When we compared these 5‐DAP SNPs with the SNPs previously reported for maize kernels at 15 DAP (Fu *et al*., 2013), we found that 308 058 SNPs (46.41% of 5 DAP and 55.15% of 15 DAP) were shared commonly in the two datasets (Figure 1a). Note that the SNPs from 15 DAP were concatenated from two analytical sources: 49 256 were determined using the MaizeSNP50 BeadChip (Li *et al*., 2012); and 509 322 were called from the RNA‐seq reads. As another SNP quality control measure, we compared the concordant rates of the 5‐ and 15‐DAP SNPs and found that the 5‐DAP SNPs shared 97.26 and 94.71% concordance with, respectively, the BeadChip‐ and RNA‐seq‐derived 15‐DAP SNPs (Figure 1b).

Prior to conducting an eQTL analysis, and with the goal of increasing mapping power, we broadened the SNP density by combining the 5‐DAP and 15‐DAP SNP datasets—this resulted in a total of 914 330 SNPs covering 28 280 genes, 3 105 more genes than were covered in the previously reported SNP data for 15‐DAP kernels. Among the SNPs used in the present study, 89.73% were located in gene regions (Figure S1a), a reasonable distribution considering that the SNPs were identified from gene expression data collected for each line of a large association population. The average number of SNPs per gene was 32 (Figure S1b). To prepare the dataset for association analysis, we examined the expression levels for all covered genes and eliminated those for which the population median expression level was zero, thereby retaining 27 103 covered genes that were expressed at 5 DAP. Considering that maize kernel is a complex structure and that a temporal‐morphological staging in one inbred line would not necessarily correlate with the same staging in another, we explored the developmental consistency of the inbred lines at 5 DAP. Genes were ranked by average expression in the inbred lines, and the correlation of average expression with each sample's expression of the top 1 000 genes was used as an indicator of consistency. Among all 282 inbred lines, 185 (65.6%) showed very strong correlation (*r *≥ 0.8) and 86 (30.5%) strong correlation (0.6 ≤ *r *< 0.8; Evans, 1996), indicating that most of the lines staged well together (Table S2). The left inbred lines not correlated well should not produce false‐positives in the following eQTL study, but might decrease detection power.

Association analysis was conducted with a mixed linear model (MLM) using the expression level data for each inbred line for each of these 27 103 genes at 5 DAP and the 776 254 integrated SNPs that had a minor allele frequency (MAF) greater than 0.05. Significantly associated SNPs were then selected using a Benjamini–Hochberg (BH) method with false discovery rate (FDR) under 0.05 (*P *< 2.6E‐6). The subsequent definition of eQTL regions based on the significant SNPs from the association analysis used a previously described approach (Fu *et al*., 2013). Briefly, when at least three significant SNPs were clustered at a genomic locus with no entailed SNP pair being more than 5 kb apart, these SNPs then were deemed as a candidate eQTL. In the case where a single gene was associated with more than one candidate eQTL, and provided that two candidate eQTLs had LD (*r* ^2 ^> 0.1), the less significant eQTL was then removed; if two candidate eQTLs had the same eQTL association strength (*P*‐value), then the eQTL with the larger joint effect was retained.

In total, this identified a total of 22 966 eQTLs associated with the expression of 18 377 genes in 5‐DAP maize kernels (Figure 1c; Table S3). The average length of these eQTLs was 4 086 bp, and they were comprised of an average of 13 SNPs. Recalling that these were mostly from RNA‐seq analysis, it was not surprising that most of the 5‐DAP eQTLs (70%) shared overlap with genic regions (37.4% in exons and 32.6% in introns). We also divided the eQTLs into two types following a previously published eQTL analysis of maize using 20 kb as a cut‐off to define ‘local eQTLs’ versus ‘distant eQTLs’ (Fu *et al*., 2013): 11 858 (51.6%) were local eQTLs, 11 108 (48.4%) were distant eQTLs. Taking the gene *ZAG2* (GRMZM2G160687) as an example, a local eQTL region was defined by 25 significantly associated SNPs within the locus (Figure 1d). Haplotype analysis based on the most strongly associated SNP in a candidate eQTL region targeting this gene defined two haplotypes in the maize association panel population, and there were two distinct *ZAG2* expression patterns that characterized these separate haplotypes (Figure 1e,f), thereby providing an illustration of the power of eQTL analysis to identify active regulatory sequences functioning in the inbred lines of the association panel.

### **Early kernel development‐related genes identified by integration of expression quantitative trait loci, quantitative trait transcript and genome‐wide association studies analyses**

**Figure 2:**
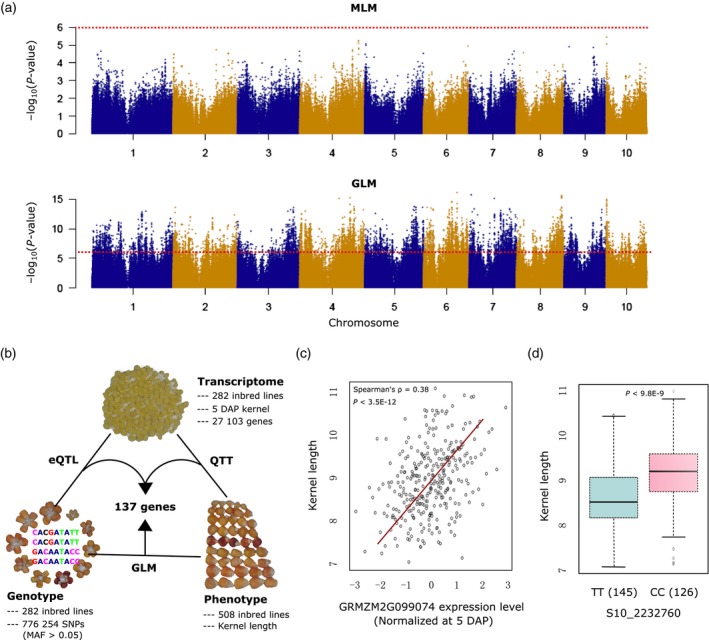
Identification of kernel length‐related genes from integrated analysis of expression quantitative trait loci (eQTL), quantitative trait transcript (QTT) and genome‐wide association studies (GWAS) analyses. (a) GWAS signals of kernel length using different association models: mixed linear model (MLM; top) and general linear model (GLM; bottom). The dashed horizontal lines represent the significant *P*‐value cut‐off (1.29E‐6). (b) Illustration of the integrative approach used to identify genes related to kernel length at 5 days after pollination (DAP). The combination of eQTL and QTT reveals kernel length‐related genes at the expressional level, and then these genes were filtered by considering the GWAS signals. MAF, minor allele frequency. (c) Correlation of the expression level of GRMZM2G099074 with kernel length. (d) Comparison of kernel length between the two haplotypes based on the most significantly associated SNP of GRMZM2G099074. The kernel length data were used from previous studies (Liu *et al*., 2016).

We next conducted QTT analysis to explore genes related to kernel length that are expressed in the 5‐DAP kernels. This analysis employed a linear regression model to characterize relationships between published kernel length data for each inbred line in the association panel (Liu *et al*., 2016) and the expression data for each of the 18 377 genes that were associated with eQTLs in each inbred line of the population. Using a cut‐off value of *P *< 0.05 and expression level of population median > 1, 905 of the genes associated with eQTLs were deemed as significant QTTs. Separately, we conducted GWAS with a MLM using the kernel length phenotype data for each inbred line and the aforementioned 776 254 SNPs. As is common with MLM‐based association analysis for complex traits in maize populations, no significant SNPs were found for kernel length, even under a loose cut‐off (1.3E‐6; Figures 2a, top, and S2; Yang *et al*., 2014). We therefore used an alternative—a general linear model (GLM)—to conduct association analysis, which revealed 12 159 significantly associated SNPs covering 3 627 genes (Figure 2a, bottom). However, as GLM‐based association studies are well known to have high false‐positives rates, particularly when the population structure is not controlled (Larsson *et al*., 2013), we retained only the genes that were common to both the GLM and the significant QTT results (Figure 2b). We thus identified 137 genes that are putatively related to kernel length at 5 DAP (Table S4). Notably, 43 (31.39%) of these genes are located in previously reported QTL regions for kernel length (Table 1).

**Table 1:** List of selected kernel length‐related genes at 5 DAP

Gene	Functional annotation	eQTL			QTT		GWAS		QTL ref.
		SNP (A1/A2)	Mean exp (A1/A2)	*P*‐value	Cor	*P*‐value	Mean KL (A1/A2)	*P*‐value	
GRMZM2G143782	mab23; Speckle‐type POZ	S6_93485430 (G/A)	13.2/32.5	< 2.2E‐16	0.33	2.1E‐04	8.7/9.2	1.0E‐07	(Li *et al*., 2013a)
GRMZM2G446426	mads52; MADS‐box transcription factor	S3_38744838 (C/T)	18.2/8.0	1.6E‐12	−0.33	2.2E‐04	8.7/9.2	1.7E‐08	(Wang *et al*., 2009; Li *et al*., 2013a)
GRMZM2G161545	Ubiquitin‐conjugating enzyme	S4_146090569 (G/C)	8.5/5.1	2.2E‐16	−0.30	1.0E‐03	8.8/9.4	5.5E‐07	(Li *et al*., 2013a)
GRMZM2G164835	NA	S6_93657368 (C/G)	8.7/10.6	2.3E‐06	0.23	1.1E‐03	8.7/9.5	6.9E‐11	(Li *et al*., 2013a)
GRMZM2G122965	Putative kinesin member	S1_47911257 (G/C)	7.3/8.3	2.6E‐02	0.27	1.7E‐03	8.6/9.1	9.5E‐08	(Li *et al*., 2013a)
GRMZM2G165746	Putative mitochondrial substrate/solute carrier	S3_60257567 (A/C)	21.0/11.2	1.6E‐12	0.28	2.3E‐03	9.0/8.6	5.8E‐07	(Li *et al*., 2013a)
GRMZM2G144615	F‐box containing protein	S3_9041378 (C/T)	21.6/17.5	6.3E‐15	−0.26	2.7E‐03	8.7/9.3	9.9E‐09	(Wang *et al*., 2009; Liu *et al*., 2017)
GRMZM2G300924	ereb170; AP2/EREBP transcription factor	S5_189817208 (C/A)	2.8/3.9	2.0E‐02	0.25	3.1E‐03	8.7/9.4	1.2E‐08	(Li *et al*., 2013a)
GRMZM2G123355	Glycosyltransferase 6	S4_191222267 (G/T)	13.7/21.7	4.3E‐07	0.23	4.6E‐03	8.8/9.5	4.5E‐09	(Wang *et al*., 2009; Li *et al*., 2013a)
GRMZM2G037177	NA	S7_169965607 (G/T)	101.6/86.1	6.2E‐09	−0.25	4.8E‐03	8.8/9.3	1.2E‐06	(Veldboom *et al*., 1994; Wang *et al*., 2009; Li *et al*., 2013a)
GRMZM2G093035	NA	S6_13921812 (G/A)	3.8/7.0	7.9E‐08	0.25	4.9E‐03	8.8/9.3	5.6E‐07	(Li *et al*., 2013a)
GRMZM2G067555	NA	S4_198308777 (A/C)	4.3/2.2	1.6E‐12	−0.23	5.3E‐03	8.7/9.3	1.7E‐07	(Wang *et al*., 2009; Li *et al*., 2013a)
GRMZM2G148867	Putative glutaredoxin	S1_201782255 (A/G)	10.3/8.4	1.6E‐12	−0.23	5.6E‐03	8.7/9.1	1.1E‐06	(Li *et al*., 2013a)
GRMZM2G154029	HLH DNA‐binding protein	S9_121002306 (C/T)	13.0/34.6	1.2E‐12	0.25	6.5E‐03	8.7/9.4	5.2E‐08	(Li *et al*., 2013a; Liu *et al*., 2017)
GRMZM2G176133	50S ribosomal protein L33	S6_158610330 (T/C)	39.3/34.1	6.0E‐05	−0.23	6.5E‐03	8.6/9.2	4.4E‐10	(Wang *et al*., 2009)
GRMZM2G001648	NA	S4_23004680 (G/A)	3.4/34.4	7.4E‐11	0.24	7.3E‐03	8.8/9.6	3.9E‐07	(Li *et al*., 2013a)
GRMZM2G092925	Protein HVA22	S1_198967213 (C/T)	4.1/16.8	1.7E‐12	0.23	7.8E‐03	8.7/9.5	1.4E‐09	(Li *et al*., 2013a)
GRMZM2G043943	Pectinesterase	S6_160526879 (A/G)	9.3/24.8	2.2E‐07	0.21	8.0E‐03	8.8/9.4	5.1E‐07	(Wang *et al*., 2009)
GRMZM2G058900	NA	S6_93993703 (A/G)	11.6/8.6	< 2.2E‐16	−0.22	8.1E‐03	8.8/9.3	3.8E‐07	(Li *et al*., 2013a)
GRMZM2G082855	pzb01110; Leucine‐rich repeat receptor‐like protein	S9_24080734 (T/G)	7.1/4.7	1.1E‐11	0.20	8.7E‐03	9.0/8.5	7.9E‐08	(Liu *et al*., 2017)
GRMZM2G126808	hb45; Homeobox protein	S1_29544058 (C/A)	12.2/24.8	4.9E‐14	0.22	8.9E‐03	8.7/9.4	1.3E‐09	(Wang *et al*., 2009; Liu *et al*., 2017)
GRMZM2G012126	NA	S1_29263923 (G/C)	13.5/5.7	< 2.2E‐16	0.19	1.1E‐02	9.1/8.6	4.7E‐09	(Wang *et al*., 2009; Liu *et al*., 2017)
GRMZM2G180622	Sarcoplasmic reticulum histidine‐rich calcium‐binding	S3_28621143 (G/A)	13.9/5.1	1.6E‐12	0.20	1.2E‐02	9.0/8.5	1.4E‐07	(Wang *et al*., 2009; Li *et al*., 2013a)
GRMZM2G147966	NA	S7_117728836 (C/T)	22.1/28.1	1.9E‐02	0.21	1.2E‐02	8.7/9.4	1.3E‐07	(Li *et al*., 2013a)
GRMZM5G874568	NA	S6_91797273 (C/G)	2.9/34.6	1.6E‐12	0.21	1.3E‐02	8.7/9.3	2.1E‐09	(Li *et al*., 2013a)
GRMZM2G139650	Phytoene dehydrogenase‐like	S5_101336590 (A/G)	12.0/18.5	1.6E‐12	−0.20	1.4E‐02	9.0/8.5	5.8E‐07	(Li *et al*., 2013a)
GRMZM2G353785	Ribosomal subunit	S7_147108286 (A/G)	9.2/5.2	1.6E‐12	0.20	1.5E‐02	9.0/8.5	2.9E‐07	(Liu *et al*., 2017)
GRMZM2G154394	NA	S4_5399845 (T/C)	7.1/9.1	3.7E‐07	−0.22	1.7E‐02	8.9/8.4	1.3E‐06	(Li *et al*., 2013a)
GRMZM2G333875	Putative kinase	S6_88865767 (T/C)	8.6/14.1	7.7E‐08	0.17	2.1E‐02	8.7/9.3	1.0E‐08	(Li *et al*., 2013a)
GRMZM2G000481	Putative tRNA synthetase	S4_34371927 (G/A)	3.3/7.2	1.6E‐12	0.21	2.2E‐02	8.7/9.3	2.7E‐10	(Li *et al*., 2013a)
GRMZM2G069146	ereb115; AP2/EREBP transcription factor	S7_141174723 (G/C)	37.8/17.1	2.1E‐12	0.17	2.3E‐02	9.1/8.6	1.3E‐06	(Liu *et al*., 2017)
GRMZM2G551402	Putative ubiquitin protease	S4_201441462 (T/C)	14.0/16.1	1.2E‐04	0.16	2.3E‐02	8.6/9.1	8.9E‐08	(Wang *et al*., 2009; Li *et al*., 2013a)
GRMZM2G053083	NA	S7_147098815 (G/C)	37.3/57.3	5.2E‐14	−0.15	2.5E‐02	9.0/8.5	9.1E‐07	(Liu *et al*., 2017)
GRMZM2G104449	NA	S4_200716340 (C/T)	8.5/9.6	3.9E‐05	−0.19	2.8E‐02	8.7/9.1	9.4E‐07	(Wang *et al*., 2009; Li *et al*., 2013a)
GRMZM2G166873	bZIP transcription factor	S8_162663083 (T/G)	14.7/15.6	4.2E‐02	0.19	2.9E‐02	8.6/9.1	1.2E‐06	(Veldboom *et al*., 1994; Wang *et al*., 2009)
GRMZM2G034598	Beta‐hexosaminidase	S6_34455108 (C/G)	16.6/21.9	1.2E‐10	−0.19	3.1E‐02	9.0/8.5	4.2E‐08	(Li *et al*., 2013a)
GRMZM2G352607	Lipid‐binding protein	S9_44283747 (C/T)	7.7/5.7	3.3E‐11	−0.19	3.2E‐02	8.8/9.5	3.2E‐08	(Li *et al*., 2013a)
GRMZM2G070716	NADH‐ubiquinone oxidoreductase B16.6 subunit	S1_25369971 (G/A)	15.5/27.5	1.6E‐12	0.16	3.3E‐02	8.7/9.2	1.4E‐07	(Wang *et al*., 2009; Liu *et al*., 2017)
GRMZM2G157536	NA	S6_155435074 (C/G)	13.1/21.8	2.3E‐06	0.17	3.8E‐02	8.7/9.2	1.1E‐06	(Veldboom *et al*., 1994; Wang *et al*., 2009)
GRMZM2G102657	Glycosyltransferase‐related family protein	S9_132170051 (C/T)	1.4/8.4	1.6E‐12	0.19	4.1E‐02	8.7/9.3	1.6E‐07	(Chen *et al*., 2016)
GRMZM5G899300	bub3; Mitotic checkpoint	S9_20608662 (T/C)	42.4/50.2	5.0E‐08	0.15	4.4E‐02	8.7/9.1	9.8E‐07	(Liu *et al*., 2017)
GRMZM2G009045	NA	S7_125138530(C/A)	5.0/9.1	4.2E‐04	0.14	4.8E‐02	8.7/9.2	3.3E‐07	(Li *et al*., 2013a)
GRMZM2G172322	gsr1; Glutathione reductase	S1_12985707 (C/G)	10.3/13.8	1.8E‐12	−0.15	4.9E‐02	9.0/8.5	1.1E‐06	(Wang *et al*., 2009)

All of the SNP names begin with ‘S’ and then followed by the chromosome number and the genomic position (AGPv3) separated with ‘_’; A1 and A2 represent the two alleles of SNP.Cor, correlation; eQTL, expression quantitative trait loci; Exp, expression; GWAS, genome‐wide association studies; KL, kernel length; QTT, quantitative trait transcript; Ref., reference; SNP, single nucleotide polymorphism.

A Gene Ontology (GO) analysis of the 137 genes identified in our integrative eQTL, QTT and GWAS analysis indicated that cell‐cycle‐related processes were enriched among our putative kernel length‐related genes (*P *= 2.0E‐4; Figure S3). The top‐ranked kernel‐related transcript (from the QTT analysis) was GRMZM2G099074; the closest homolog (55% identity of protein sequences; E = 3.0E‐118) to this gene in Arabidopsis is *embryo defective 1379* (*AtEMB1379* or *AtNSE1*, AT5G21140), a gene with established functions in regulating the cell cycle at the G2/M stage and in endo‐reduplication (Li *et al*., 2017). The correlation between the expression of GRMZM2G099074 and kernel length was high (Spearman's *ρ* = 0.38, *P *< 3.5E‐12; Figure 2c). Moreover, a haplotype analysis based on the most strongly associated SNP in an eQTL region for GRMZM2G099074 defined two haplotypes, and there were obvious and significant differences in kernel length between these two haplotypes (Figure 2d).

Our identification of a strong association between GRMZM2G099074 and kernel length, as well as the over‐representation of cell cycle regulation‐related genes indicated that tight control of gene expression relating to cell cycle regulation during the early phase of kernel development may be critical in determining the eventual size of mature kernels. Supporting this idea, we found an eQTL overlapping a gene putatively encoding a mitotic spindle checkpoint protein *MAD2* (GRMZM2G112072) was associated with the expression of a predicted chromatin remodeling protein (GRMZM2G138125). The homolog of GRMZM2G138125 in rice is known to be essential for syncytial development at the early stages of endosperm development (Hara *et al*., 2015), strongly suggesting a similar role for this gene in maize kernel development.

### **Comparison of expression quantitative trait loci from 5 DAP and 15 DAP**

**Figure 3:**
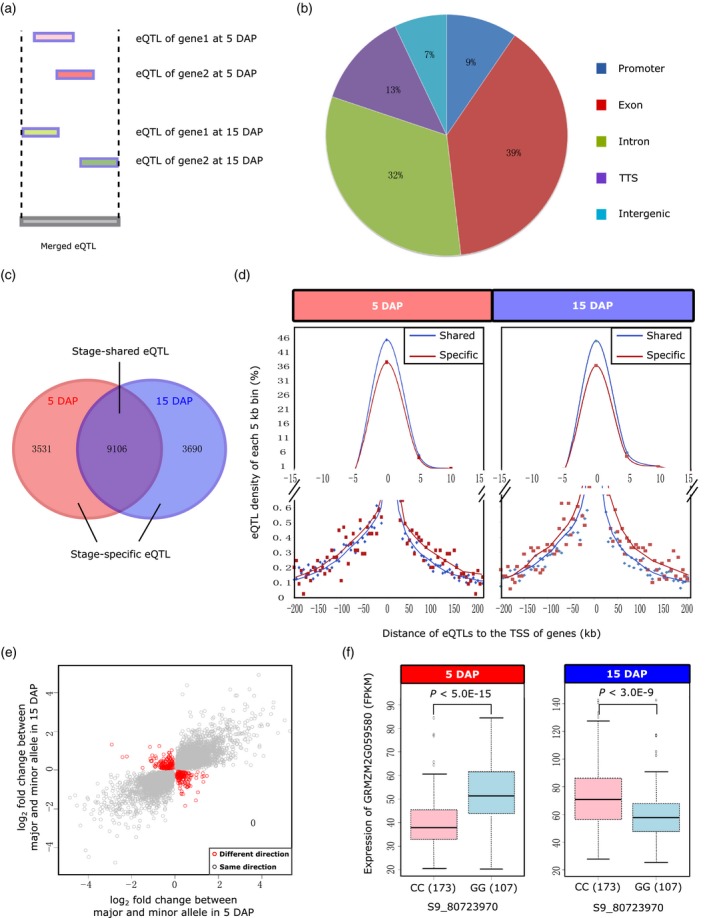
Integration and comparison of the expression quantitative trait loci (eQTLs) identified from 5 days after pollination (DAP) and 15 DAP maize kernels. (a) Schematic diagram showing the integration of the eQTL regions by retaining the maximum region for any intersecting eQTLs of the two stages. (b) The genomic distribution of the merged eQTL dataset. (c) Venn diagram to show the number of stage‐shared and stage‐specific eQTLs. (d) Positional distribution of the stage‐shared and stage‐specific eQTLs relative to the transcriptional start site (TSS) of their associated genes. The *x*‐axis shows the distance of eQTLs to the annotated TSS of their associated genes. The *y*‐axis represents the eQTL density per 5‐kb bin. Red dots and lines highlight the distribution pattern of stage‐specific eQTLs, and blue ones represent stage‐shared eQTLs. (e) The allelic effect of stage‐shared eQTLs. Each point represents an eQTL‐gene association that was significant in both stages. For each eQTL, the lead SNP with the most significant association was chosen as a representative example. The axes show the expression divergence (fold change) between the major and minor alleles of the associated genes at both stages. The eQTLs with inverse regulatory effects between the two stages are shown in red. (f) One eQTL example with allelic effect to its associated gene. The expression levels of the two alleles of GRMZM2G059580 are shown for both 5 DAP and 15 DAP, and inverse regulatory trends are presented with box plots.

We next compared the eQTLs from 5 DAP with the eQTLs for 15 DAP. To maximize the comparability between the two eQTL datasets, all the eQTL regions identified from 5 DAP and 15 DAP were merged by retaining the maximum genomic region for any intersecting eQTLs (Figure 3a). We thus obtained 16 327 merged eQTL regions, with an average length of 4 418 bp and comprised of an average of 35 SNPs, that were associated with 18 377 and 18 872 genes for the 5‐DAP and 15‐DAP kernels, respectively (Table S5). Consistent with the data source for the SNPs (based on RNA expression), most of the merged eQTLs (71%) shared overlap with genic regions (39% in exons and 32% in introns), and 22% of these eQTLs shared overlap with UTR sequences [here defined as within 1 kb of the transcription start site (TSS) or termination site]; the remaining 7% eQTLs fell into intergenic regions (Figure 3b).

In total, 9 106 eQTLs (accounting for 55.8% of the merged eQTL dataset) were commonly detected in developing kernels of both stages (‘stage‐shared’ eQTLs). There were also 3 531 ‘5 DAP‐specific’ eQTLs and 3 690 ‘15 DAP‐specific’ eQTLs (Figure 3c). Close inspection of the physical distribution of the stage‐shared versus the stage‐specific eQTLs revealed a fascinating trend: for a given gene with an associated eQTL, the distance to the associated gene's TSS was larger for the stage‐specific eQTLs than for the stage‐shared eQTLs (Figure 3d). A possible implication here is that the eQTLs that exert their regulatory function within a relatively narrower temporal/developmental window may be relatively more likely to use distant mechanisms. It will be interesting to examine the presence/absence of similar trends in other plant genomes as they undergo various developmental sequences. GO functional category analysis of genes associated with the 5‐DAP‐specific eQTLs were enriched for plant responses to stimulus, while the targets of 15‐DAP‐specific eQTLs were enriched for nutrient reservoir activity, a finding consistent with the known physiology of the grain filling occurring at 15 DAP. Targets of stage‐shared eQTLs were enriched for development‐related processes like multicellular organismal development, anatomical structure development and organelle organization (Figure S4).

An analysis of the regulatory effects of stage‐shared eQTLs on individual target genes at 5 DAP versus 15 DAP indicated that 324 out of 9 106 stage‐shared eQTLs exhibited ‘inverse regulatory activities’ (Figure 3e). For instance, an eQTL with two alleles (‘CC’ or ‘GG’) was significantly associated with the expression of GRMZM2G059580 (a gene that encodes the elongation factor 1‐gamma) at both 5 DAP and 15 DAP, but the expression of this gene of each particular allele was inverted between the two developmental stages: lines with the ‘CC’ allele had relatively lower GRMZM2G059580 expression at 5 DAP, whereas lines with this same allele had relatively higher GRMZM2G059580 expression at 15 DAP (Figure 3f). Similar phenomena have been reported in eQTL studies of humans for tissue‐specific inverse regulatory effects for a single allele (Fu *et al*., 2012). It is notable that the proportion of such eQTLs in humans was similar to what we observed in maize kernel development. When we closely examined the distance of these eQTLs to their associated genes, we found that fully 323 out of the 324 such eQTLs were local eQTLs (Table S6). Note that this is over‐represented relative to the distribution pattern of the whole set of the stage‐shared eQTLs, for which 51.1% of eQTLs were local (chi‐square test, *P *< 2.2E‐16). The observation of eQTLs with inverse effects at 5 DAP and 15 DAP may indicate the different regulatory mechanisms of gene expression under distinct developmental stages, but we cannot rule out the possibility that the inverse effects might be caused by expression change affected by different growth conditions when collecting samples for the two datasets.

### **Expression quantitative trait loci associated with multiple genes and a candidate** ^**epi**^ **eQTL for kernel traits**

In our study, about 90% of the eQTLs were associated with fewer than three targets (91.4% at 5 DAP and 90.1% at 15 DAP), and more than 50% eQTLs were associated with only one gene. We defined eQTLs associated with more than three targets as ‘multi‐target eQTLs’; at this threshold, 1 083 and 1 265 multi‐target eQTLs identified for 5 DAP and 15 DAP, respectively (Table S7). There were 53 and 79 stage‐specific multi‐target eQTLs for 5 DAP and 15 DAP, respectively (Tables S8 and S9). GO analysis showed that the target genes of the 5‐DAP‐specific multi‐target eQTLs were enriched for functions relating to response to abiotic stimulus as well as generation of precursor metabolites and energy (Table S10), while genes associated with the 15‐DAP‐specific multi‐target eQTLs were enriched for nutrient reservoir activity (Table S11).

**Figure 4:**
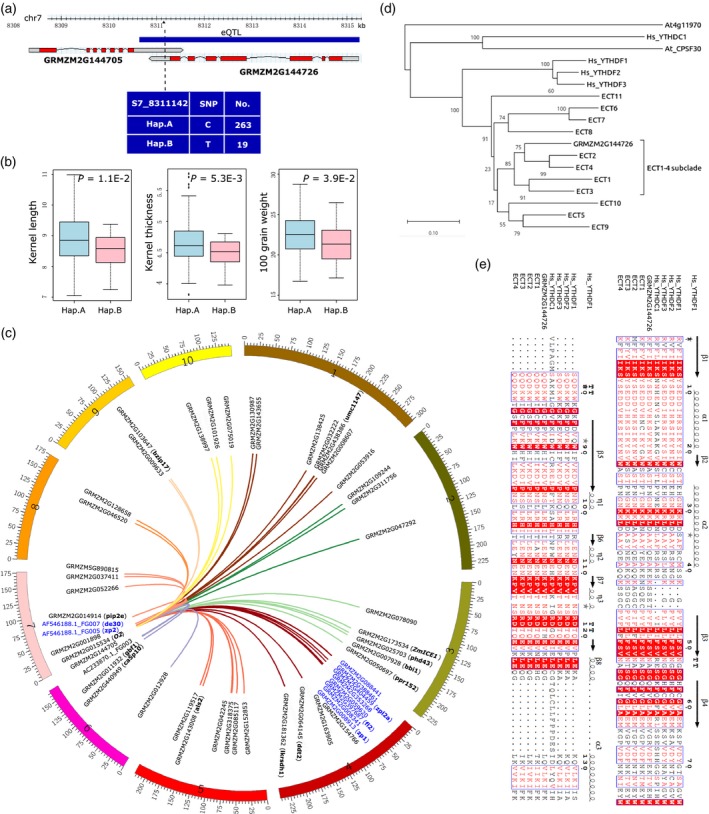
A proposed ^epi^eQTL associated with multiple genes at 15 days after pollination (DAP). (a) The genomic position of one 15‐DAP‐specific expression quantitative trait loci (eQTL; chr7: 8310530…8315224) associated with 53 genes. Information for the two haplotypes based on the most significantly associated single nucleotide polymorphism (SNP; S7_8311142) is highlighted. (b) The phenotypic divergence (*t*‐test) of the two haplotypes for kernel length, kernel thickness and 100 grain weight. The kernel phenotypic data were used from previous studies (Liu *et al*., 2016). (c) The genomic distribution of all the 53 target genes associated with this eQTL. Genes that are annotated as functional or putative zein genes are highlighted in blue. (d) The phylogenetic tree of GRMZM2G144726 with other YTH domain‐containing genes in both human and Arabidopsis. GRMZM2G144726 was grouped with ECT1‐4 as the ‘ECT1‐4‐subclade’. (e) Multiple sequence alignment of the YTH domain for the ECT1‐4‐subclade genes with the YTH‐domain‐containing genes in human. The boxes highlight the conserved regions among them; the amino acids with red background are highly conserved sites.

We found one apparently extremely important multi‐target eQTL located on chromosome 7 (chr7:8310530…8315224) that is specific at 15 DAP and associated with 53 genes spread across all 10 chromosomes of the maize genome. This eQTL spans the GRMZM2G144726 locus, which is located within a previously reported QTL region for kernel weight (Figure 4a; Liu *et al*., 2017), and was highly expressed in endosperm, embryo and whole kernel during kernel development based on published expression atlas (Figure S5; Chen *et al*., 2014). Haplotype analysis based on the most significant SNP (S7_8311142) in this eQTL showed that there are two haplotypes for it in the association panel. There were significant differences in the kernel phenotypes: one of the two haplotypes had longer and thicker kernels and had higher 100 grain weights (‘large kernel haplotype’; Figure 4b). Among the 53 associated genes, 10 are genes for zein proteins, which are the most abundant storage proteins in the maize endosperm (Figure 4c; Thompson and Larkins, 1994). It was also highly notable that the *opaque‐2* gene (*O2*, GRMZM2G015534), the long‐studied and well‐known regulator of zein gene expression (Zhang *et al*., 2015), was among the 53 targets of this eQTL. Likewise, the basic helix‐loop‐helix (bHLH) gene *ZmICE1* (GRMZM2G173534)*—*which encodes a protein that strongly interacts with O11, a central regulator of endosperm development and nutrient metabolism that directly regulates the expression of *O2* on the upstream (Feng *et al*., 2018)—was also among the 53 associated genes. Beyond this, *ZmICE1*,* O2* as well as the 10 zein genes had significantly higher expression levels in the large kernel haplotype (Figure S6).

Fascinatingly, the GRMZM2G144726 locus covered by this eQTL encodes a homolog of the Arabidopsis *ECT2* gene (AT3G13460), which encodes a very recently characterized ‘reader’ protein for the epi‐transcriptomic mark N6‐methyladenosine (m^6^A; Arribas‐Hernández *et al*., 2018; Scutenaire *et al*., 2018; Wei *et al*., 2018; Figure 4d,e). For context, in a process conceptually similar to DNA methylation, m^6^A marks on mRNA can be dynamically written, erased and read, and these marks are known to affect gene regulation. ECT2 is one of the first reported m^6^A readers in Arabidopsis; it is a YTH‐domain family protein required for normal trichome (Arribas‐Hernández *et al*., 2018; Scutenaire *et al*., 2018; Wei *et al*., 2018) and leaf development (Arribas‐Hernández *et al*., 2018). It has been revealed that m^6^A in Arabidopsis mainly occurs in the RRACH motif (Shen *et al*., 2016), while a recent study found that ECT2 may bind m^6^A/RNA through a URUAY motif in Arabidopsis (Wei *et al*., 2018). Pursuing the idea that the eQTL overlapping the GRMZM2G144726 locus may relate to some epi‐transcriptomic mechanism controlling kernel development, we searched for both RRACH and URUAY motifs among the transcripts of the 53 target genes. We found that the enrichment of URUAY motif in the transcript regions was slightly significant in the 53 genes (79.2% versus 69.7% in background, *P *= 0.04) compared with that of RRACH motif (98.1% versus 99.2% in background, *P *= 0.29), although both motifs were found in high frequency of presence. We did not find significant enrichment in the 3’‐UTRs of their transcripts for both URUAY motif (*P *= 0.15) and RRACH motif (*P *= 0.15; Table S12). Further experiments are needed to determine the m^6^A motif in all the 53 genes that were significantly differentially expressed between the two haplotypes (Table S13).

The function of Arabidopsis ECT2 as an m^6^A reader depends on the presence of the YTH‐domain (Arribas‐Hernández *et al*., 2018; Scutenaire *et al*., 2018; Wei *et al*., 2018). To explore the possibility that the SNPs in the eQTL region overlapping GRMZM2G144726 locus may change the amino acid sequence of the YTH domain thereby affecting its function, we annotated all the 48 SNPs in this eQTL region and found only three of them located in the putative YTH domain of GRMZM2G144726 (Table S14). These three SNPs are under LD with the most significantly associated SNP (S7_8311142) of GRMZM2G144726 (Figure S7a). Two of them are synonymous variants, while the other one is a non‐synonymous variant, which leads to the change of Glu466 to Asp466, a non‐conserved amino acid of the YTH domain (Figure S7b,c; Arribas‐Hernández *et al*., 2018), suggesting that it is unlikely that these variants will change the m^6^A/RNA‐binding ability of the YTH domain of GRMZM2G144726. Given that GRMZM2G144726 was significantly differentially expressed in the two haplotypes (Table S13), we speculate that a yet unknown mechanism impacting the transcript levels of GRMZM2G144726 may exist to influence its function in kernel development in the population of inbred lines.

These data suggest that our quantitative genetics analysis has identified an ^epi^eQTL for a master epi‐transcriptomic regulator of kernel development (GRMZM2G144726, henceforth *ZmECT2*). The possibility that a major yield trait is regulated epi‐transcriptomically is an exciting idea, and our transcriptome analysis for the 5‐DAP and 15‐DAP kernels in the association panel lines can serve as the initial dataset for exploring these ideas. Nevertheless, experiments that specifically monitor the marked and unmarked forms of target transcripts will be needed to guide future investigations into (among other questions) the distribution of m^6^A‐binding motifs on target transcripts, the rate of target mRNA turnover, the cellular localization(s) and population(s) of marked versus unmarked target transcripts, and the effects of genetically manipulating *ZmECT2* on all of the aforementioned topics.

## Experimental Procedures

### **Plant germplasm, RNA preparation and Illumina sequencing**

The total number of lines used in the study of 5‐DAP maize kernels is 282, of which 268 were from the 368 inbred lines used in the previous 15‐DAP eQTL analyses (Yang *et al*., 2010; Fu *et al*., 2013) and the other 14 of which are newly added temperate inbred lines. All 282 of the lines were planted in Hainan province (China) in 2014 in one‐row plots using an incompletely randomized block design with three replicates, which is the same as previous 15‐DAP designs except that the planting location of 15‐DAP samples was Jingzhou, Hubei province (China) in 2010. Eight ears in each block were self‐pollinated, and 10–20 seeds from 3–4 ears in each block were collected at 5 DAP. The collected seeds of the three replications were mixed for extraction of total RNA. Total RNA was extracted using a RNAprep pure kit for plants (TIANGEN, Beijing, China) according to the product instruction. Total mRNA was accumulated using oligo(dT), and library construction for Illumina protocols was conducted using manufacturer‐specified methods (Illumina, San Diego, USA). Pair‐end sequencing with 125 bp was carried out for each sample using the Illumina HiSeq 2500 sequencing platform.

### Reads mapping

Sequenced reads were cleaned after removing adapters and low‐quality sequences, and reads greater than 50 bp were kept for further analysis. The cleaned reads were mapped to maize B73 AGPv3 reference genome using TopHat2 (Kim *et al*., 2013) with the following key parameters: ‘‐i 5 ‐I 60000 –library‐type fr‐unstranded ‐r 250 –mate‐std‐dev 60 –no‐novel‐juncs –microexon‐search’. The insertion size (−*r*) and standard deviation (–mate‐std‐dev) of paired reads were estimated using ‘CollectInsertSizeMetrics.jar’ plugin in Picard tools (http://picard.sourceforge.net). Only reads that could be uniquely mapped to the genome were retained for further analysis.

### Quantification of gene expression levels

Read counts for annotated genes (AGPv3, Ensembl release 27) were calculated using *GenomicFeatures* and *GenomicAlignments* packages (Lawrence *et al*., 2013). The *DESeq2* package (Love *et al*., 2014) was used to normalize the read counts among samples and to calculate the FPKM value of each gene. The genes with a population median expression level > 0 were kept for association analysis. To avoid effects from outliers and non‐normality of gene expression values when conducting the association analysis, the qqnorm function in R (Ihaka and Gentleman, 1996) was used to normalize the expression value of each gene.

### Single nucleotide polymorphism calling and imputation

GATK (version v3.6; McKenna *et al*., 2010) was applied for SNP calling. The parameters were set based on GATK best practices pipeline for RNA‐seq (DePristo *et al*., 2011; Van der Auwera *et al*., 2013). Briefly, SNPs were called separately for each sample by setting the minimum phred‐scaled confidence threshold for calling variants 20 (‐stand_call_conf 20) and then filtered based on Fisher strand bias < 30.0 and quality by depth > 2.0. Then the SNPs generating from different samples were merged and the missing information for some loci was recalled using HaplotypeCaller by adding the parameter ‘‐gt_mode GENOTYPE_GIVEN_ALLELES’. During the procedure of SNP calling, Picard tools were used to reorder and sort reads in mapping files, and mark duplicates. Known indel information for maize was downloaded from the Ensembl Plants website (http://ftp://ftp.ensemblgenomes.org/pub/plants/release-27/vcf/zea_mays/zea_mays.vcf.gz). SNPs with heterozygote rate > 10% or missing rate > 60% were filtered out before imputation. Heterozygous genotypes (0/1) were recorded and replaced as missing (./.). The MAF of each SNP was then calculated, and those with MAF < 2% were also discarded.

BEAGLE v4.1 (Browning and Browning, 2016) was used to impute missing genotypes because of its good performance under high levels of missing rate for low‐coverage sequencing data (Pasaniuc *et al*., 2012; Swarts *et al*., 2014). We applied BEAGLE with the strategy suggested in the CONVERGE studies (CONVERGE consortium, 2015), which was found to have superior imputation accuracy relative to other approaches (Davies *et al*., 2016). We first imputed all sites without a reference panel and then imputed with a reference panel (Bukowski *et al*., 2018). The two sets of imputation results were merged by replacing the former with the latter if extant. Heterozygous genotypes after imputation and the imputed genotypes, which had been recorded as heterozygous sites in the SNP calling procedures, were both masked as missing (./.).

The genotypes called in the present study were compared with those called from the maize 15‐DAP study (Fu *et al*., 2013). A concordance rate was calculated based on the SNPs from the same maize inbred lines. The SNPs of two datasets were merged for further analysis. For overlapped SNP loci, the genotypes called from the present study (5 DAP) were retained. The *VariantAnnotation* package (Obenchain *et al*., 2014) was used to annotate SNPs and categorize them into five regions (5’‐UTR, CDS, Intron, 3’‐UTR and Intergenic region) according to their genomic locations. The *GenomicFeatures* package (Lawrence *et al*., 2013) was used to read the ‘gtf’ format file for gene annotation.

### Population structure, kinship estimations and hidden factor analysis for gene expression profiles

Before association analysis, principal components and kinship matrices were calculated based on 236 205 SNPs for which there were none missing and which had a MAF > 0.05 using TASSEL v5 (Bradbury *et al*., 2007). The top three principal components were used as population structure, and the ‘Normalized_IBS’ method was used during the kinship estimation.

The hidden factors of gene expression variability were analyzed using PEER (Stegle *et al*., 2010), which uses a Bayesian framework to account for complex non‐genetic factors. The top three principal components (population structure) were used as covariates into the model during analysis. We used the prior parameters *a*_α _= 10^−7 ^G and *b*_α _= 10^−1 ^G in the noise precision distribution, where *G* is the total number of genes. Finally, 10 PEER hidden factors were included in the following association analyses to increase power and interpretability.

### Association analysis and identification of expression quantitative trait loci

The association analyses between SNPs and gene expression levels were conducted using a MLM implemented in TASSEL v5 (Bradbury *et al*., 2007). The top three principal components and 10 hidden factors were used as covariates, and the kinship matrix was used as the variance‐covariance matrix of the random effects. A BH method was applied to control FDR at level *α* = 0.05. The method to identify eQTLs was similar to the previous study (Fu *et al*., 2013). Adjacent SNPs with distance less than 5 kb were grouped as a cluster, and the clusters with at least three significant SNPs were considered as candidate eQTLs. If two candidate eQTLs of a single gene were under LD (*r* ^2 ^> 0.1), the less significant eQTL was dropped. If two candidate eQTLs had the same eQTL association significance (*P*‐value), the eQTL with the less joint effect was removed.

### Identification and display of multi‐target expression quantitative trait loci

For each eQTL region, we calculated the number of its targets at both developmental stages. The eQTLs were defined as multi‐target eQTLs if they were associated with more than three genes at either 5 or 15 DAP. The links between the eQTL and their targets were visualized using Circos software (Krzywinski *et al*., 2009).

### Association analysis for kernel length

Association analysis for kernel length was also conducted using TASSEL v5 (Bradbury *et al*., 2007). For the MLM, the top three principal components were used as covariates, and the kinship matrix was used as the variance‐covariance matrix of the random effects. For the GLM, no covariates or random effects were included.

### Linear regression model for detection of quantitative trait transcripts in kernel length

We used the following linear regression model to identify genes correlated with kernel length:

$$E_{\mathrm{ij}}=u_{j}+\alpha_{j}{\text{KL}}_{i}+\sum_{k=1}^{3}\beta_{\mathrm{jk}}{\text{PC}}_{\mathrm{ik}}+\sum_{k=1}^{N}\gamma_{\mathrm{jk}}{\text{HF}}_{\mathrm{ik}}+\delta_{\mathrm{ij}}$$

where, *E*_*ij*_ is the expression level of gene *j* in inbred line *i*,* u*_*j*_ is the regression intercept, KL_*i*_ denotes the kernel length of inbred line *i*, PC_*ik*_ (1≤ *k *≤ 3) presents the value of the *k*‐th principal component value of the genotype for inbred line *i*, HF_*ik*_ (1≤ *k *≤ *N*) denotes the value of the *k*‐th hidden factor of gene expression profile for inbred line *i*,* N* is the top hidden factors under consideration, δ_*ij*_ is the error term, and, α_*j*_, β_*jk*_, γ_*jk*_, are the regression coefficients for kernel length, the *k*‐th principal component, and the *k*‐th hidden factor, respectively. In the model, we considered the top two hidden factors, which had broad effects on gene expression variation in the population. For each gene, if α_*j*_ was significantly deviated from 0, gene *j* was considered to be related to kernel length. We used *P *< 0.05 as a cut‐off, and defined that gene *j* was upregulated with the phenotype if α_*j*_ > 0 and downregulated if α_*j*_ < 0.

### Gene ontology enrichment analysis

The GO enrichment analysis for genes was carried out using the online toolkit agriGO (Du *et al*., 2010). The minimum number of mapping entries was set to 5, and Fisher's exact tests were used to examine the significance of accumulation against the background of the corresponding whole genome (AGPv3, Ensembl release 30). The *P*‐values were then adjusted using a Benjamini–Yekutieli method (FDR < 0.05).

### Motif enrichment analysis

To search the distribution of motifs on the full sequence of mRNAs or 3’‐UTR regions, regular expressions in the Perl programming language were used to match the published motifs on the cDNA sequences. The regular expression of RRACH motif is [GA][GA]AC[TAC], while the regular expression of URUAY is T[GA]TA[TA]. If one gene has multiple transcripts, we define a motif in its mRNA/3’‐UTR when any transcript of this gene contains a motif in its mRNA/3’‐UTR. After counting the number of genes containing motifs, the hypergeometric function was used to calculate the statistical significance of motif enrichment.

## Accession numbers

The RNA sequencing data for the 282 maize inbred lines in 5‐DAP kernels have been submitted to the NCBI Sequence Read Archive (SRA; http://www.ncbi.nlm.nih.gov/sra/) with the BioProject ID PRJNA413629 or study accession ID SRP119998. The processed gene expression abundance data, including reads counts as well as FPKM values, could be accessed through NCBI Gene Expression Omnibus (GEO; https://www.ncbi.nlm.nih.gov/geo/) with the series entry GSE110315. The SNP dataset was submitted to the European Variation Archive (EVA; https://www.ebi.ac.uk/eva) with the accession number as PRJEB24974. All the procedures and scripts for reads mapping, gene expression analysis and SNP calling can be found in the following website: https://github.com/Pang-Junling/maize-5DAP-eQTL.

## Author contributions

JZ, GW and YF designed and supervised the project. JP, JF and NZ contributed equally to this paper as first authors. NZ, DS, JW and HZ prepared the materials and performed the experiments. JP, JF, CH and TF performed the data analysis. JP, JF, NZ and XZ prepared the manuscript. JZ and GW contributed to the revision of the manuscript. All authors read and approved the final manuscript.

## Conflict of interest

The authors declare that they have no conflict of interests.

## Supporting information


**Figure S1.** Statistics of the 914 330 SNPs combined from data of both 5‐DAP and 15‐DAP maize kernels.

**Figure S2.** QQ‐plot for the GWAS results of kernel length using MLM.

**Figure S3.** GO analysis for the 137 genes that associated with kernel length.

**Figure S4.** GO analysis for the targets of stage‐shared and stage‐specific eQTLs.

**Figure S5.** The expression levels of GRMZM2G144726 in different tissues of maize based on published data.

**Figure S6.** The differential expression of *ZmICE1*,* O2* as well as the 10 zein genes in the two haplotypes defined by the ^epi^eQTL.

**Figure S7.** Display of the SNPs in the eQTL region overlapping GRMZM2G144726 locus and their relationship to the putative YTH domain of GRMZM2G144726.

**Table S1.** The mapping statistics of the RNA sequencing data from 282 samples

**Table S2.** The correlation between the average gene expression levels in the association population with individual expression levels in each inbred lines of the top 1000 genes highly expressed

**Table S3.** The 22 966 eQTLs for 18 377 genes in 5‐DAP maize kernels

**Table S4.** List of kernel length related genes at 5 DAP

**Table S5.** List of the merged eQTL regions and their corresponding targets at both 5 DAP and 15 DAP

**Table S6.** The 324 genes associated with eQTLs with inverse regulatory effects

**Table S7.** List of the multi‐target eQTLs at both 5 DAP and 15 DAP

**Table S8.** List of the 53 5‐DAP‐specific multi‐target eQTLs

**Table S9.** List of the 79 15‐DAP‐specific multi‐target eQTLs

**Table S10.** GO analysis of the genes associated with 5‐DAP‐specific multi‐target eQTL

**Table S11.** GO analysis of the genes associated with 15‐DAP‐specific multi‐target eQTL

**Table S12.** The statistics of the ‘URUAY’ and ‘RRACH’ motifs on transcripts of the targets of GRMZM2G144726

**Table S13.** Differential expression of GRMZM2G144726 and its 53 target genes in 15‐DAP kernels between the two haplotypes of GRMZM2G144726

**Table S14.** Annotation of the SNPs in the eQTL region overlapping GRMZM2G144726
