# Supplementary material for: Kernel size‐related genes revealed by an integrated eQTL analysis during early maize kernel development
Source: Plant J. 2019 Jan 25;98(1):19–32. doi: 10.1111/tpj.14193 (PMC6850110; doi:10.1111/tpj.14193)
Supplement: Supplementary file 1 — Figure S1. Statistics of the 914 330 SNPs combined from data of both 5‐DAP and 15‐DAP maize kernels. [file TPJ-98-19-s001.pdf]

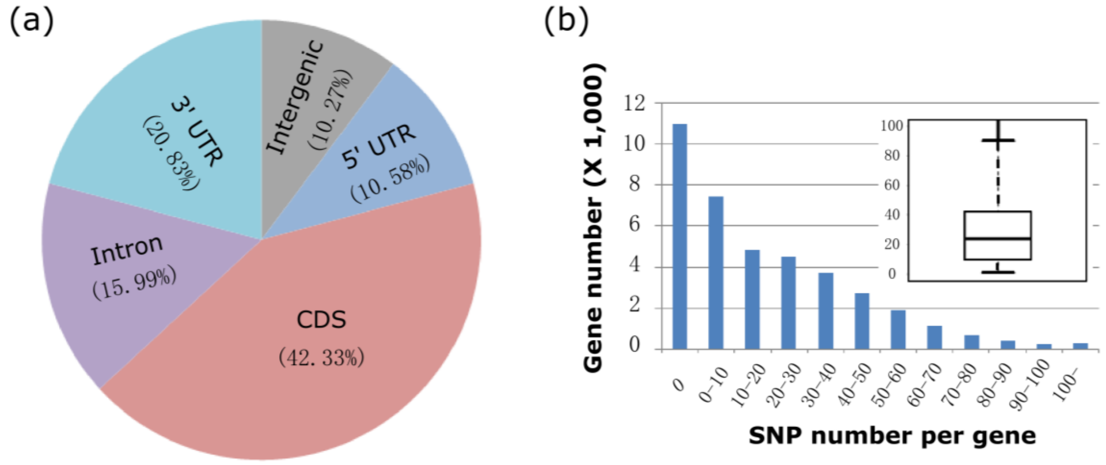


**Figure S1. Statistics of the 914,330 SNPs combined from data of both 5 DAP and 15 DAP maize kernels.** (a) The genomic distributions of the SNPs. (b) The number of SNPs located on each gene locus.
