# Supplementary material for: Kernel size‐related genes revealed by an integrated eQTL analysis during early maize kernel development
Source: Plant J. 2019 Jan 25;98(1):19–32. doi: 10.1111/tpj.14193 (PMC6850110; doi:10.1111/tpj.14193)
Supplement: Supplementary file 2 — Figure S2. QQ‐plot for the GWAS results of kernel length using MLM. [file TPJ-98-19-s002.pdf]

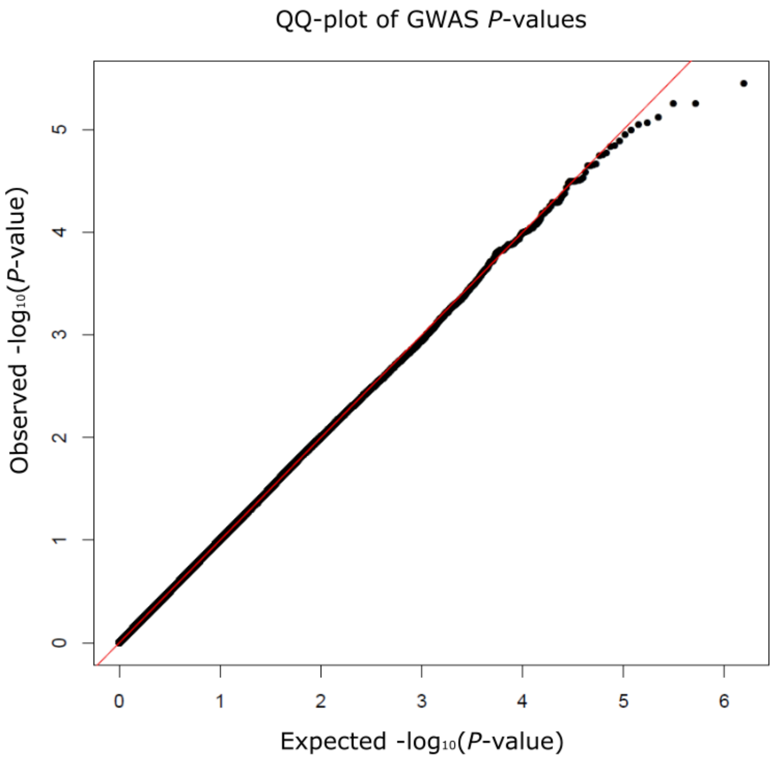

Figure S2. QQ-plot for the GWAS results of kernel length using MLM. The expected and observed *P*-values for each SNPs in the association analysis were shown in x-axis and y-axis, respectively. The red line shows the condition y=x.
