# Supplementary material for: Kernel size‐related genes revealed by an integrated eQTL analysis during early maize kernel development
Source: Plant J. 2019 Jan 25;98(1):19–32. doi: 10.1111/tpj.14193 (PMC6850110; doi:10.1111/tpj.14193)
Supplement: Supplementary file 3 — Figure S3. GO analysis for the 137 genes that associated with kernel length. [file TPJ-98-19-s003.pdf]

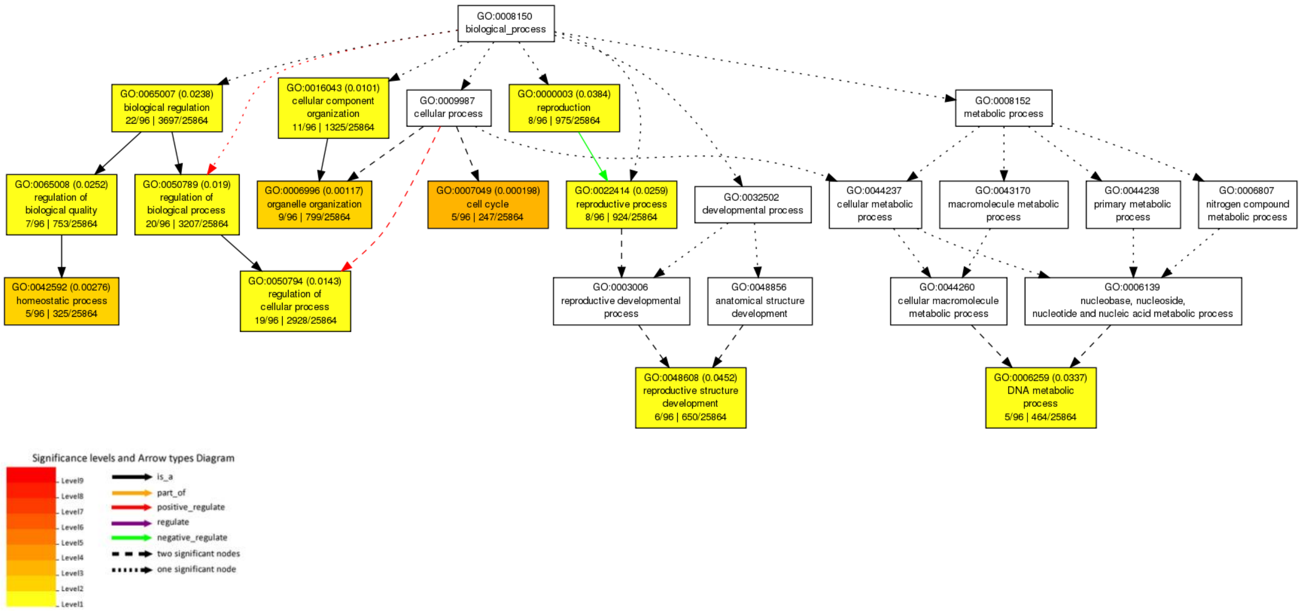

Figure S3. GO analysis for the 137 genes that associated with kernel length. *P*-value for each significant item is shown in the parentheses.
