# Supplementary material for: Kernel size‐related genes revealed by an integrated eQTL analysis during early maize kernel development
Source: Plant J. 2019 Jan 25;98(1):19–32. doi: 10.1111/tpj.14193 (PMC6850110; doi:10.1111/tpj.14193)
Supplement: Supplementary file 4 — Figure S4. GO analysis for the targets of stage‐shared and stage‐specific eQTLs. [file TPJ-98-19-s004.pdf]

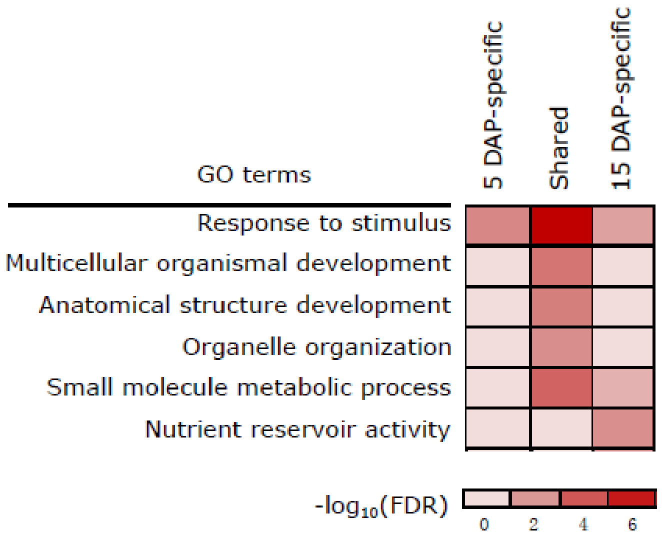

Figure S4. GO analysis for the targets of stage-shared and stage-specific eQTLs. The significance of accumulation was shown in colors.
