# Supplementary material for: Kernel size‐related genes revealed by an integrated eQTL analysis during early maize kernel development
Source: Plant J. 2019 Jan 25;98(1):19–32. doi: 10.1111/tpj.14193 (PMC6850110; doi:10.1111/tpj.14193)
Supplement: Supplementary file 5 — Figure S5. The expression levels of GRMZM2G144726 in different tissues of maize based on published data. [file TPJ-98-19-s005.pdf]

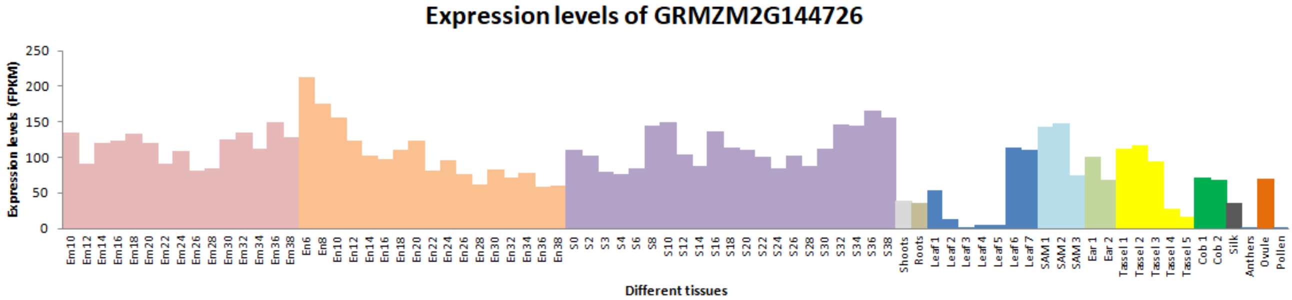

Figure S5. The expression levels of GRMZM2G144726 in different tissues of maize based on published data. The expression data was accessed through previous results (Chen et al., 2014). Em: embro; En: endosperm; S: whole seed; SAM: shoot apical meristem.
