# Supplementary material for: Kernel size‐related genes revealed by an integrated eQTL analysis during early maize kernel development
Source: Plant J. 2019 Jan 25;98(1):19–32. doi: 10.1111/tpj.14193 (PMC6850110; doi:10.1111/tpj.14193)
Supplement: Supplementary file 6 — Figure S6. The differential expression of ZmICE1, O2 as well as the 10 zein genes in the two haplotypes defined by the epieQTL. [file TPJ-98-19-s006.pdf]

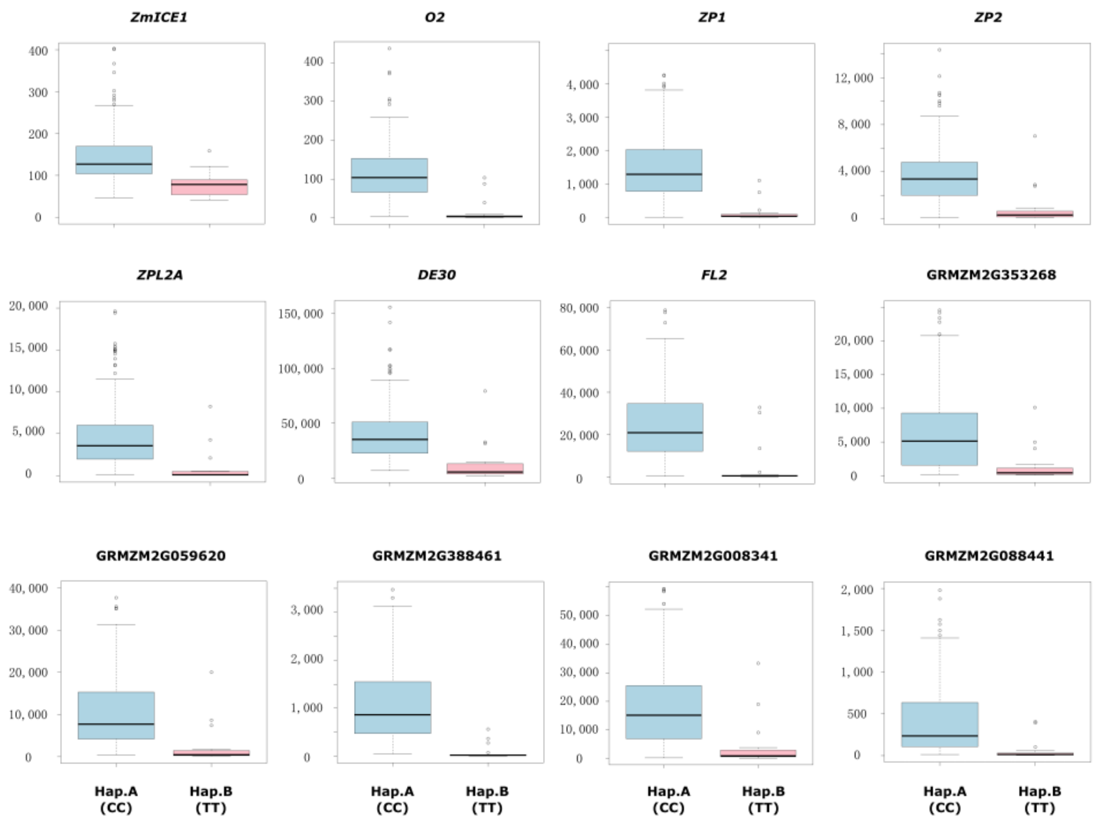

Figure S6. The differential expression of *ZmICE1*, *O2* as well as the 10 zein genes in the two haplotypes defined by the ^{epi}eQTL. The haplotype A (Hap.A) is the large kernel haplotype, while haplotype B (Hap.B) corresponds to relative small kernels in the population.
