# Supplementary material for: Kernel size‐related genes revealed by an integrated eQTL analysis during early maize kernel development
Source: Plant J. 2019 Jan 25;98(1):19–32. doi: 10.1111/tpj.14193 (PMC6850110; doi:10.1111/tpj.14193)
Supplement: Supplementary file 7 — Figure S7. Display of the SNPs in the eQTL region overlapping GRMZM2G144726 locus and their relationship to the putative YTH domain of GRMZM2G144726. [file TPJ-98-19-s007.pdf]

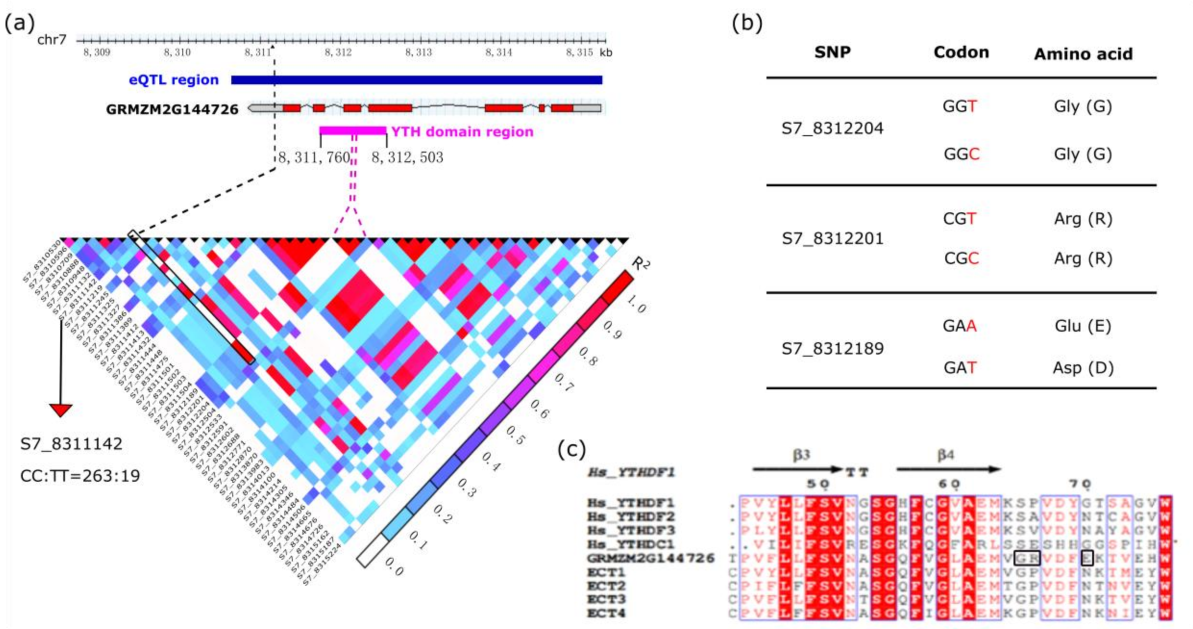

Figure S7. Display of the SNPs in the eQTL region overlapping GRMZM2G144726 locus and their relationship to the putative YTH domain of GRMZM2G144726. (a) The genomic position of the YTH domain of GRMZM2G144726 as well as the SNPs in the eQTL region. Four SNPs are highlighted with lines, among which the most significantly associated SNP (S7_8311142) was located in the 3′ UTR of GRMZM2G144726 and the other three (S7_8312189, S7_8312201 and S7_8312204) locate in the putative YTH domain region. The four SNPs are with high LD, which highlighted in a black box. (b) The detailed information of the three SNPs in the YTH domain. The red color highlights the substitution of the SNPs in codons of the three amino acids showed in the right-most column. (c) The positions in the YTH domain of the three amino acids showed in (b), which are highlight with black boxes. The conserved amino acids were marked with red.
