# Supplementary material for: Kernel size‐related genes revealed by an integrated eQTL analysis during early maize kernel development
Source: Plant J. 2019 Jan 25;98(1):19–32. doi: 10.1111/tpj.14193 (PMC6850110; doi:10.1111/tpj.14193)
Supplement: Supplementary file 22 [file TPJ-98-19-s022.doc]

**Figure S1. Statistics of the 914,330 SNPs combined from data of both 5 DAP and 15 DAP maize kernels.** (a) The genomic distributions of the SNPs. (b) The number of SNPs located on each gene locus.

**Figure S2. QQ-plot for the GWAS results of kernel length using MLM.** The expected and observed *P*-values for each SNPs in the association analysis were shown in x-axis and y-axis, respectively. The red line shows the condition y=x.

**Figure S3. GO analysis for the 137 genes that associated with kernel length.** *P*-value for each significant item is shown in the parentheses.

**Figure S4. GO analysis for the targets of stage-shared and stage-specific eQTLs.** The significance of accumulation was shown in colors.

**Figure S5. The expression levels of GRMZM2G144726 in different tissues of maize based on published data.** The expression data was accessed through previous results (Chen et al., 2014). Em: embro; En: endosperm; S: whole seed; SAM: shoot apical meristem.

**Figure S6. The differential expression of *ZmICE1*, *O2* as well as the 10 zein genes in the two haplotypes defined by the ^epi^eQTL.** The haplotype A (Hap.A) is the large kernel haplotype, while haplotype B (Hap.B) corresponds to relative small kernels in the population.

**Figure S7. Display of the SNPs in the eQTL region overlapping GRMZM2G144726 locus and their relationship to the putative YTH domain of GRMZM2G144726.** (a) The genomic position of the YTH domain of GRMZM2G144726 as well as the SNPs in the eQTL region. Four SNPs are highlighted with lines, among which the most significantly associated SNP (S7_8311142) was located in the 3′ UTR of GRMZM2G144726 and the other three (S7_8312189, S7_8312201 and S7_8312204) locate in the putative YTH domain region. The four SNPs are with high LD, which highlighted in a black box. (b) The detailed information of the three SNPs in the YTH domain. The red color highlights the substitution of the SNPs in codons of the three amino acids showed in the right-most column. (c) The positions in the YTH domain of the three amino acids showed in (b), which are highlight with black boxes. The conserved amino acids were marked with red.

**Table S1.** The mapping statistics of the RNA sequencing data from 282 samples.

**Table S2.** The correlation between the average gene expression levels in the association population with individual expression levels in each inbred lines of the top 1,000 genes highly expressed.

**Table S3.** The 22,966 eQTLs for 18,377 genes in 5-DAP maize kernels.

**Table S4.** List of kernel length related genes at 5 DAP.

**Table S5.** List of the merged eQTL regions and their corresponding targets at both 5 DAP and 15 DAP.

**Table S6.** The 324 genes associated with eQTLs with inverse regulatory effects.

**Table S7.** List of the multi-target eQTLs at both 5 DAP and 15 DAP.

**Table S8.** List of the 53 5-DAP-specific multi-target eQTLs.

**Table S9.** List of the 79 15-DAP-specific multi-target eQTLs.

**Table S10.** GO analysis of the genes associated with 5-DAP-specific multi-target eQTL.

**Table S11.** GO analysis of the genes associated with 15-DAP-specific multi-target eQTL.

**Table S12.** The statistics of the "URUAY" and "RRACH" motifs on transcripts of the targets of GRMZM2G144726.

**Table S13.** Differential expression of GRMZM2G144726 and its 53 target genes in 15-DAP kernels between the two haplotypes of GRMZM2G144726.

**Table S14.** Annotation of the SNPs in the eQTL region overlapping GRMZM2G144726.
